# Granulocytic Sarcoma of the Stomach Presenting as Dysphagia during Pregnancy

**DOI:** 10.1155/2011/627549

**Published:** 2011-10-20

**Authors:** Anuradha Sekaran, Santosh Darisetty, Sandeep Lakhtakia, Mohan Ramchandani, Duvuru Nageshwar Reddy

**Affiliations:** ^1^Department of Pathology Asian Institute of Gastroenterology, 6-3-661 Somajiguda, Hyderabad 500 082, India; ^2^Department of Anesthesiology Asian Institute of Gastroenterology, 6-3-661 Somajiguda, Hyderabad 500 082, India; ^3^Department of Medical Gastroenterology Asian Institute of Gastroenterology, 6-3-661 Somajiguda, Hyderabad 500 082, India

## Abstract

Granulocytic sarcoma also known as extramedullary myeloid sarcoma or chloroma is an uncommon manifestation of leukemia and presents as a deposit of leukemic cells outside the bone marrow. We report a case of a twenty-five-year-old pregnant woman who presented with progressive dysphagia and recurrent postprandial vomiting. Upper GI endoscopy had shown large flat laterally spread nodular lesions in the cardia and proximal body of stomach. Biopsies from the gastric lesion showed granulocytic sarcoma of the stomach. Concurrent peripheral and bone marrow picture was suggestive of acute myeloid leukemia (AML–M4). There is limited reported literature on granulocytic sarcoma of the stomach. Concurrent gastric granulocytic sarcoma involving cardia and AML in pregnancy has not been reported till date.

## 1. Introduction

Granulocytic sarcoma also known as chloroma or extramedullary myeloid tumor is a collection of leukemic cells outside the bone marrow. These tumors occur in 3–8% of acute nonlymphoid leukemia. The common sites affected by it are skin, lymph nodes, central nervous system, and reproductive organs. Involvement of gastrointestinal tract is relatively rare with small bowel being the commonest site. 

We report a case of gastric myeloid sarcoma in a young female at 17 weeks of gestation presenting with dysphagia, epigastric pain, and vomiting.

## 2. Case Report

**Figure 1:**
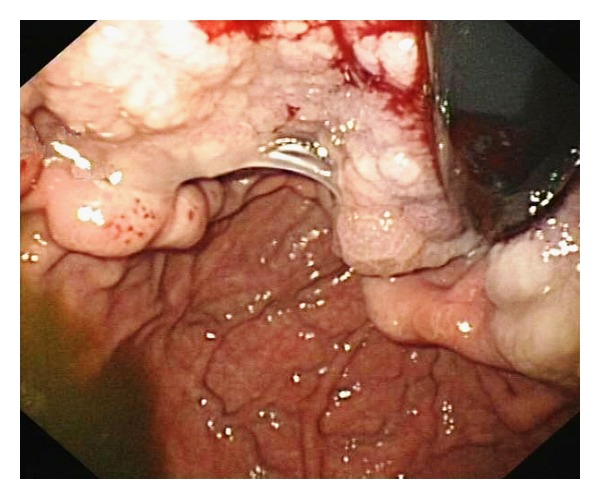
Upper GI endoscopy showing large area of nodular gastric mucosa, extending from GE junction to surrounding body.

**Figure 2:**
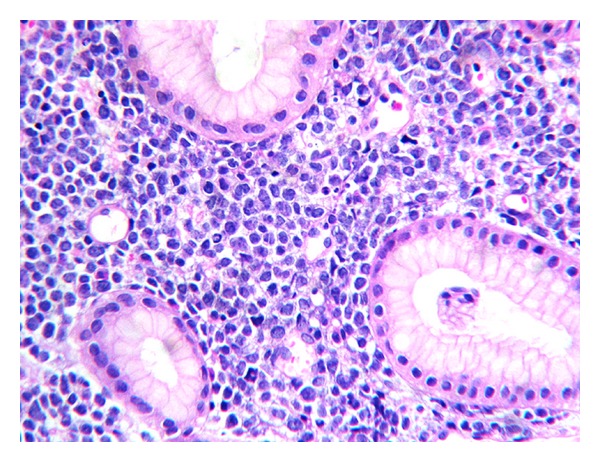
H&E, 40×. Unremarkable foveolae and glands with expansion of lamina propria by atypical round cells, admixed with granulocytic precursors.

**Figure 3:**
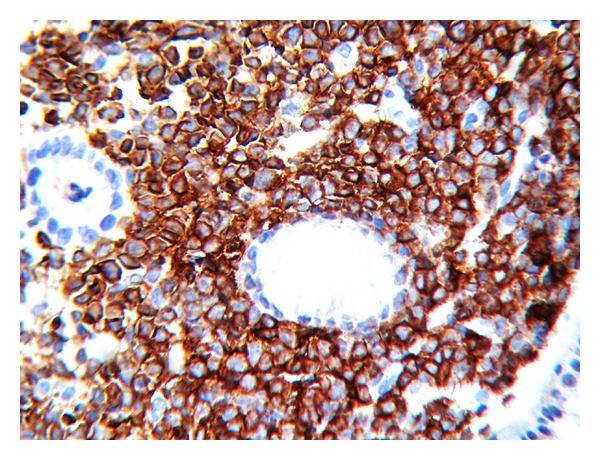
H&E, 400×. Sheets of atypical round cells IHC-CD 117 marker, 400×. Intense positivity in all atypical cells for immature myeloid marker CD 117.

**Figure 4:**
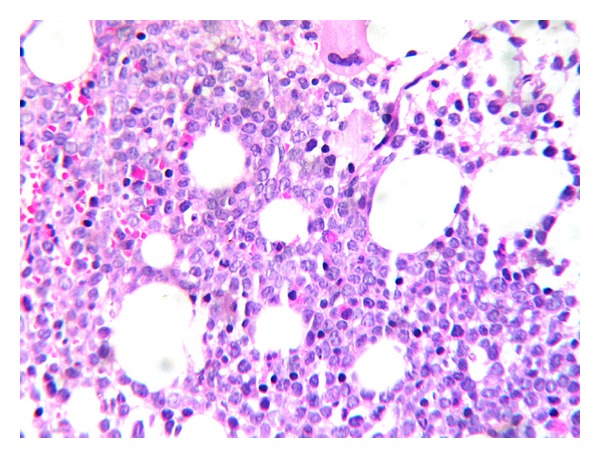
Trephine bone marrow biopsy, H&E, 400×. Sheets of blasts replacing normal marrow elements.

A 25-year-old woman with 17 weeks of gestation (G3P2) presented with progressive dysphagia, epigastric discomfort, persistent postprandial vomiting, and weight loss for one month. Initial investigations were within normal limits. Upper GI endoscopy showed thickened nodular friable mucosa at cardia of stomach extending into the posterior wall (Figure 1). Multiple deep biopsies from gastric cardia showed normal mucosal glands with expansion of lamina propria by infiltrates of atypical cells, intermixed with granulocytic precursor cells (Figure 2). Immunohistochemistry panel showed that the cells were negative for CD20, CD5, CD3, CD10, and cytokeratin markers, with intense positivity for immature myeloid markers (myeloperoxidase, CD34, and CD117) (Figure 3), consistent with granulocytic sarcoma of the stomach. A week later she developed fever with WBC count of 34,800 cells per cu mm. A bone marrow biopsy (Figure 4) and flow cytometry analysis showed features consistent with acute myeloid leukemia (AML-M4) with blast count of 77%. Pregnancy was terminated.

## 3. Discussion

Granulocytic sarcoma is a rare malignant solid tumor composed of primitive precursors of the granulocytes deposited outside the bone marrow. It was called “chloroma” due to its green color. Rappaport renamed it “granulocytic sarcoma” because the color varied depending on the concentration of the pigmented enzyme (myeloperoxidase). It is more common in children than adults and often occurs concurrently with AML. They rarely present with relapse of leukemia, in blast crisis of myeloproliferative disorder, as leukemic transformation in myelodysplastic syndrome [1]. Granulocytic sarcoma in the Gastrointestinal (GI) tract arises from nests of hematopoietic cells [2]. The commonest site of involvement is small bowel [3] followed by colon, appendix, biliary tract, and stomach [4].

 The endoscopy findings can vary from normal to mucosal nodularity or mass lesions. It may precede hematologic leukemia by months or years [5]. Tissue biopsies, bone marrow biopsy, immunohistochemistry, and cytogenetics assist to diagnose chloroma. It is common in patients with M2, M4, or M5 class of French-American British (FAB) classification. 

Systemic intensive chemotherapy is the first line of treatment [6]. Our patient is taking chemotherapy and is doing well on followup. This subject was different from other reported cases of gastric chloroma. She presented with dysphagia in pregnancy and the diagnosis of gastric chloroma preceded the detection of AML involving peripheral blood and bone marrow. To our knowledge, this is the first case of the AML presenting as sarcoma of stomach in pregnancy. Involvement of cardia of stomach has also not been reported earlier.

## Acronyms

AML: Acute Myeloid Leukemia
FAB: French-American-British
GIT: Gastrointestinal Tract
